# A genetransfer strategy based on durum wheat–*Aegilops comosa* amphiploid top-crossed with hexaploid wheat results in elimination of D chromosomes led to the production of homoeologous M(D) substitutions and translocations

**DOI:** 10.1186/s12870-025-07950-4

**Published:** 2026-01-08

**Authors:** Péter Kovács, András Farkas, Edina Türkösi, Klaudia Kruppa, Éva Szakács, Kitti Szőke-Pázsi, Norbert Hidvégi, Péter Mikó, Andrea Gulyás, Éva Darkó, Mahmoud Said, László Ivanizs, Eszter Gaál, István Molnár

**Affiliations:** 1 Hungarian Research Network (HUN-REN), Centre for Agricultural Research, Agricultural Institute, Martonvásár, 2462 Hungary; 2 Institute of Experimental Botany of the Czech Academy of Sciences, Centre of Plant Structural and Functional Genomics, Olomouc, 77900 Czech Republic; 3 Field Crops Research Institute, Agricultural Research Centre, 9 Gamma Street, Giza, 12619 Egypt; 4 Hungarian University of Agriculture and Life Sciences, Gödöllő, 2100 Hungary

**Keywords:** Interspecific hybridization, *Aegilops comosa*, Wheat–*Aegilops* introgression, *In situ* hybridization, Chromosome elimination

## Abstract

**Background:**

Domestication and nearly eight thousand years of cultivation have resulted in a narrow genetic base, which hinders the identification of effective allele combinations and hampers breeding progress under changing environmental conditions. The gene portfolio can be extended using a crossing strategy providing favourable conditions for the formation of wheat × alien chromosome addition, substitution and translocation lines. To utilize the gene pool of *Aegilops comosa*, the present study applied durum wheat–*Ae. comosa* amphiploids top-crossed (TC) by hexaploid wheat (Mv9kr1*ph1b*), a strategy exploiting monosomic conditions of D- and M-genomes in TC_1_ to form cytogenetic stocks.

**Results:**

While the expected genome structure of amphiploid and TC_1_ generations was confirmed, consecutive in situ hybridization using D- and M-genomic, as well as DNA repeat probes on 52 TC_2_F_1_ lines, showed significant elimination of not only M- but also D-genome chromosomes. Differences in the elimination frequency seemed to be related to chromosome size, with an opposite tendency for the two genomes, as shorter M chromosomes (1M, 4M, 6M) were retained more frequently, while the shorter D chromosomes (1D, 4D) were predominantly eliminated. D–M rearrangements within group 1, 3, 4, and 6 chromosomes were dominantly homoeologous, with 5D/5M recombinations observed at the highest frequency. Besides monosomic introgressions, disomic substitutions 2M(2D) and 7M(7D), addition 6M, and translocation T6MS·6ML-6D were selected. Morphological characterization and yield components indicated the good compensation ability of these *Ae. comosa* chromosomes for the loss of those of wheat.

**Conclusions:**

Relationships between chromosomes size and their elimination from wheat × alien hybrid progenies were discussed. Based on the high level of homoeologous recombinations and substitutions, top-crossing has proven to be an effective strategy for transferring alien chromosome segments from closely related diploid species into wheat. The newly developed wheat–*Ae. comosa* prebreeding materials represent potentially valuable genetic resources for wheat improvement.

**Supplementary Information:**

The online version contains supplementary material available at 10.1186/s12870-025-07950-4.

## Background

Bread wheat (*Triticum aestivum* L.) is cultivated on the largest area among cereal crops (220.7 million ha), yielding 791.02 million tons, making it the second highest in global cereal production after corn [1]. The allohexaploid genome structure (2n = 6x = 42, AABBDD) of bread wheat evolved by two successive interspecific hybridizations. The first hybridization, 0.8–0.3 million years ago, occurred between *T. urartu* Thumanjan ex Gandilyan (2n = 2x = 14, A^u^A^u^), the donor of the A genome, and a species similar to *Aegilops speltoides* Tausch (2n = 2x = 14, SS), the putative donor of B-genome, resulting in the formation of wild emmer wheat *T. turgidum* ssp. *dicoccoides* (Asch. & Graebn.) Thell. (A^u^A^u^BB, 2n = 4x = 28) [2, 3]. The second hybridization, approximately 11,000–8,000 years ago, between the wild emmer wheat and *Ae. tauschii* Coss. (2n = 2x = 14, DD), the donor of D-genome, led to the evolution of allohexaploid wheat [4, 5]. However, the genetic diversity of hexaploid wheat is reduced compared with that of its wild genome donors, as only a few accessions participated in the allopolyploidization event [6, 7] while domestication and thousands of years of cultivation have further decreased its variation [8–10]. Hence, it is a real challenge to develop efficient allele combinations to produce high-yielding and stress-tolerant cultivars capable of tolerating the adverse effects of global climate change. Interspecific or intergeneric hybridization is an effective approach to transfer new gene variants from wild related species to increase the genetic diversity of wheat [11–13]. The genus *Aegilops* is closely related to *Triticum* [14–18] and contains eleven diploid and twelve allopolyploid species [19]. Among the seven different genomes (C, D, M, N, S, T, and U) identified in the diploid species, the annual diploid *Ae. comosa* Sm. in Sibth. et Sm. (syn. *T. comosum* (Sm. in Sibth. et Sm.) K. Richt.) (2n = 2x = 14, genome MM) is considered as the donor of M genome found in the allotetraploid *Ae. geniculata* Roth. (2n = 4x = 28, U^g^U^g^M^g^M^g^) and *Ae. biuncialis* Vis. (2n = 4x = 28, U^b^U^b^M^b^M^b^).

The M-genomes of *Ae. comosa* and its allotetraploid hybrids contain genes suitable to improve agronomic traits of wheat, such as resistance against leaf, stripe, and stem rust [20, 21]to salinity [22–24] and drought [25, 26], and grain nutritional quality [27–29]. The useful agronomic traits of *Ae. comosa* can be utilized in breeding hexaploid wheat via interspecific crossing programs aimed to produce wheat × *Ae. comosa* disomic addition, substitution and translocation lines [21, 30–32]. The traditional backcross (BC) method [33] employs hexaploid wheat (2n = 6x = 42, AABBDD) crossed with a diploid alien species (2n = 2x = 14, XX) to produce F₁ hybrids (*n* = 4x = 28, ABDX). The haploid F₁ seedlings are subsequently treated with colchicine to induce genome doubling and obtain amphiploids (2n = 8x = 56, AABBDDXX). Amphiploids are partially fertile and can be backcrossed with the parental wheat genotypes, resulting in BC₁, BC₂, or BC₃ generations. During these backcrosses, the gradual elimination of alien chromosomes leads to the development of mono- or disomic addition and translocation lines [34–36]. In the case of *Ae. comosa*, the backcrossing method enabled the production of wheat–*Ae. comosa* disomic addition lines 2M–7M and 2D/2M translocation lines [21, 30–32].

The alternative crossing program, referred to as the “top-cross” strategy, is based on the development of genome-substituted synthetic hexaploid wheat forms in which the D-genome is replaced by a diploid alien genome [12, 37]. This hybridization program starts with a cross between durum wheat (*T. turgidum* ssp. *durum*) (2n = 4x = 28, AABB) and a diploid alien species (XX) to produce F_1_ hybrids (*n* = 3x = 21, ABX), followed by genome duplication using colchicine to develop synthetic hexaploids (2n = 6x = 42, AABBXX) [38]. Then wheat–*Aegilops* synthetic hexaploids can be top-crossed (TC) with hexaploid wheat (AABBDD), resulting in TC_1_ plants, where the alien and D-genomes are in monosomic conditions (AABB + X + D). This double monosomic condition of TC_1_ plants results in high frequency of univalents in the meiotic metaphase I. Univalents may undergo differential segregation into progeny cells, potentially leading to the formation of mono- or disomic substitution lines in later generations. Alternatively, univalents may undergo spontaneous centromeric breakage during anaphase I, giving rise to telosomes. Subsequent association between wheat and alien telosomes can result in the formation of wheat–alien Robertsonian translocations comprising two different chromosome arms fused at the centromere [2, 39]. Both euploid wheat–alien substitution lines and Robertsonian translocation lines are genetically more stable than aneuploid addition lines (2n = 44), making them suitable for evaluating the ability of alien chromosomes to compensate for the loss of corresponding wheat chromosomes [40]. Moreover, these lines are of considerable importance in wheat improvement, as they facilitate targeted gene transfer. This strategy has been employed to transfer alien chromosomes from *Haynaldia villosa* and *Ae. speltoides* into wheat [41, 42]. While disomic wheat–*Ae. comosa* substitution and translocation lines were also developed using this method [21, 30, 31, 43–45], a comprehensive analysis of the elimination of M-genome chromosomes and the frequency of intergenomic translocations has not been investigated.

The use of molecular cytogenetic methods, such as genomic in situ hybridization (GISH) of fluorescently labeled genomic DNA isolated from the alien crossing partner, allows the detection of alien chromosomes or chromosome segments in the wheat genetic background [46], as was demonstrated for the M-genome of *Aegilops* species [47]. The use of DNA repeat probes, including pSc119.2, Afa-family and 45S rDNA for fluorescence in situ hybridization (FISH) enables the identification of wheat and *Ae. comosa* chromosomes based on specific hybridization patterns [48–51].

With the aim of determining the elimination pattern of M-genome chromosomes and motivated by the need to produce wheat–*Ae. comosa* introgression lines, the main goals of the present study were to investigate the frequency of M chromosomes in a TC_2_F_1_ population originating from *T. turgidum* ssp. *durum* ‘GK Novodur’ × *Ae. comosa* MvGB1039 synthetic hexaploids top-crossed by hexaploid wheat line Mv9kr1*ph1b*. The wheat and M chromosomes were identified by sequential genomic- and fluorescence in situ hybridization (GISH and FISH) using M- and D-genomic probes and probes for DNA repeats Afa-family, pSc119.2, and pTa71 (45S rDNA), respectively. Finally, we also provide a morphological description of the new wheat–*Aegilops comosa* disomic addition, substitution, and translocation lines.

## Methods

### Plant material

**Fig. 1:**
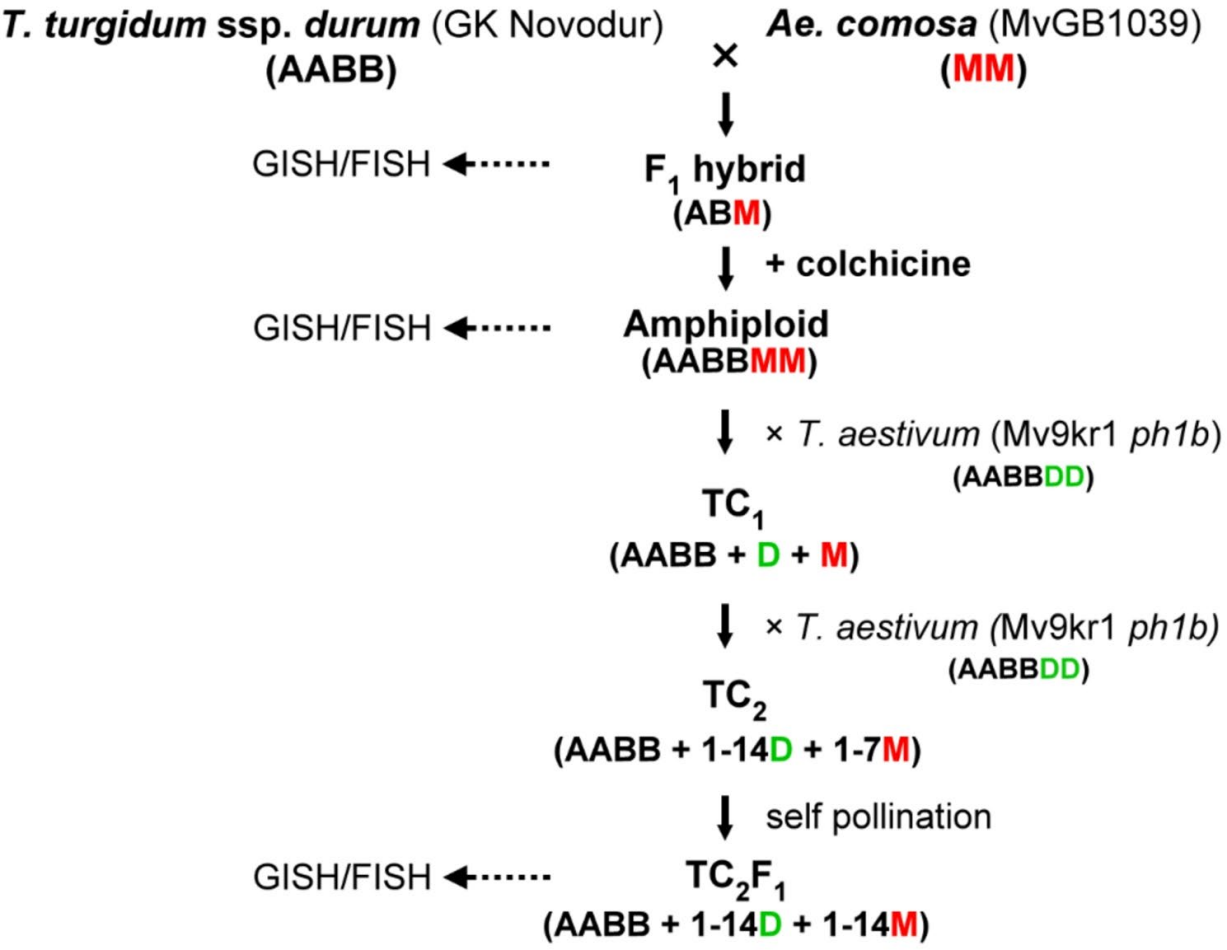
The top-cross strategy employed to facilitate the introgression of *Aegilops comosa* chromatin into bread wheat

*T. turgidum* spp. *durum* L. (2n = 4x = 28, AABB) ‘GK Novodur’ was used as maternal parent for the crosses with *Ae. comosa* (2n = 2x = 14, MM) accession MvGB1039, which was maintained in the Martonvásár Cereal Genebank, to produce durum wheat × *Ae. comosa* F_1_ hybrids. To produce durum wheat–*Ae. comosa* amphiploid (AABBMM) lines, F_1_ seeds were germinated and 7-day-old seedlings were vernalized for 6 weeks at 4 °C with a 10/14 h day/night cycle at 12 µmol m^− 2^ s^− 1^ light intensity. Vernalized seedlings were transferred into 2 L pots with a 2:1:1 mixture of garden soil, humus, and sand. F_1_ plants at the 3–4 leaf stage (Zadoks scale Z24) were removed from the soil and incubated in 0.04% (w/v) colchicine at 15 °C overnight (16 h). Then the roots were washed in tap water for 2 h, and the plants were taken back into soil pots to grow in a glasshouse (Global Glasshouse Venlo) under the conditions described by Türkösi et al. [52]. Amphiploids were top-crossed (TC) with hexaploid winter wheat (*Triticum aestivum* L.) line Mv9kr1*ph1b* containing both the recessive crossability gene *kr1* and the *ph1b* mutation (Mv9kr1*ph1b*) [53, 54] to produce TC_1,_ TC_2_, and self-pollinated TC_2_F_1_ lines, as was summarized in Fig. 1.

### Preparation of metaphase chromosome spreads

The seeds were germinated, and the mitotic division of root tip meristematic cells was synchronized using hydroxyurea and amiprophos-methyl, as described by Doležel et al. [55]. Mitotic metaphase chromosome spreads were prepared from synchronized root tip meristematic cells using the drop technique, as described by Kato et al. [56, 57] and modified by Danilova et al. [58] and Said et al. [50]. Briefly, 2 cm fragments of synchronized root tips were incubated in ice-cold 90% acetic acid for 10 min, washed three times in 70% ethanol, and stored in 70% ethanol at −20 °C. For the preparation of chromosome spreads, the root tips were washed in ice-cold Milli-Q water for 3 × 3 min, and ice cold 1× KCl buffer (75mM KCl, 7.5mM EDTA, pH 4) for 5 min. Afterwards, the root tips were incubated in 20 µl of a mixture containing 4% cellulase Onozuka R-10 (Yakult, Japan, Tokyo cat. #201069) and 1% Pectolyase Y-23 in 1× KCl buffer [58] at 37 °C for 57 min. The digested root-tip samples were treated with ice cold 400 µl TE buffer (Tris-EDTA buffer, pH 7.6), followed by three washes in ice cold 100% ethanol. Finally, 14 µl ice cold acetic acid: methanol mixture (9:1) was added, the root tips were smashed, and 7 µl of the suspension was dropped from a height of 5 cm onto glass slides and placed in a humid box until desiccation.

### Probe labeling and hybridization

Total genomic DNA (gDNA) was extracted from fresh young leaves of *Ae. comosa* accession MvGB1039 and *Ae. tauschii* accession MvGB590 using a QuickGene-Mini80 DNA isolation kit (Fujifilm, Tokyo, Japan) according to the manufacturer’s instructions. The M-genomic DNA of *Ae. comosa* was labeled with biotin (biotin-16-dUTP), while gDNA of *Ae. tauschii* (DD) was labeled by digoxigenin (digoxigenin-11-dUTP) using random priming (BioPrime™ DNA Labeling System, Thermo Fisher Scientific, Waltham, USA) and used as M- and D-genomic probes, respectively. Unlabeled gDNA of hexaploid wheat (Mv9kr1*ph1b*) was sheared and used as blocking DNA. DNA repeat probes Afa-family [59], pSc119.2 [60], and pTa71 [61] were labeled with digoxigenin-11-dUTP (Roche, Mannheim, Germany), biotin-16-dUTP (Roche, Mannheim, Germany), and a mix of biotin-11-dUTP (50%) and digoxigenin-11-dUTP (50%), respectively, by nick translation using standard kits from Roche following the manufacturer’s protocol. Digoxigenin and biotin signals were detected using anti-digoxigenin-rhodamine Fab fragments (Roche) and streptavidin-Alexa Fluor-488, respectively.

Genomic in situ hybridization (GISH) experiments were carried out as described by Molnár et al. [47]. Briefly, after the pretreatments and stringency washes, chromosomal DNA was denatured in the presence of hybridization mixture (25 µL per slide; containing 50% formamide, 2×SSC, 25% dextran sulphate, 7 ng of each M- and D-genomic probe, and 1.05 µg of blocking DNA) at 80 °C for 2 min 50 sec, and allowed to hybridize overnight at 42 °C. Following post-hybridization washes, biotin- and digoxigenin signals were detected by 10 µg/mL each of streptavidin-Alexa Fluor 488 conjugate and anti-digoxigenin-rhodamine (Molecular Probes, Waltham, USA), respectively, and the slides were counterstained with 2 µg/mL 4’−6’-diamidino-2-phenylindole (DAPI) (Amersham). After documentation of GISH results, hybridization signals were washed out (3 × 30 min. in 4xSSC Tween, 2 × 5 min 2xSSC at 25 °C) and fluorescent in situ hybridization (FISH) using DNA repeat probes pSc119.2, pTa71, and Afa-family was carried out according to Molnár et al. [47]. The results of GISH and FISH were captured using an automated Zeiss Axio Imager Z2 upright epifluorescence microscope (Carl Zeiss, Jena, Germany) equipped with a MetaSystems CoolCube 4 USB Laboratory Camera controlled by Metafer 4 (automatic metaphase image capture) and processed by ISIS (image analysis) softwares (MetaSystems, Altlussheim, Germany).

### Scoring of chromosome rearrangements and statistical analysis

Since homoeologous recombination occurs in these wheat–*Ae. comosa* hybrid derivatives, the identity of chromosomes was determined based on centromere origin. Therefore, a chromosome was considered to be of *Ae. comosa* origin, if its (peri)centromeric region was labeled with the M-genome-specific probe, even if the rest of the chromosome consisted of wheat chromatin. Chromosomes involved in intergenomic translocations were identified based on their FISH hybridization pattern as previously described by Molnár et al. [62] and Said et al. [50].

The transmission rate of each M and D homoeologous group chromosomes into the TC_2_F_1_ generation has been investigated using χ^2^-tests based on the null hypothesis that there are no differences between the theoretical and observed frequencies of each M or D chromosome (i.e., each is expected to occur at equal frequency in the population) as described by Polgári et al. [63]. Critical sample size for χ^2^-analysis was determined using MS Excel based on the following criteria: alpha (probability of Type I error) = 0.05, *df* = (number of groups)−1. Statistical associations between chromosomes (double combinations) for nonrandom elimination were investigated first with the χ^2^-test for goodness of fit.

### Morphological characterization

The morphological characterization of wheat–*Ae. comosa* disomic substitution lines 7M(7D) and 2M(2D), the disomic 6M addition line, and the disomic T6MS·6ML-6D translocation line were carried out alongside their hexaploid and tetraploid wheat parental lines, Mv9kr1*ph1b* and GK Novodur, respectively. All plants were grown under controlled glasshouse conditions in Martonvásár in 2024. The following traits were measured: plant height, number of spikes per plant, length of main spike, number of spikelets per main spike, number of grains per main spike, number of grains per plant, and thousand kernel weight (TKW) were measured on ten plants (wheat parental lines) or one plant (wheat–*Ae. comosa* cytogenetic stocks).

## Results

### Development of wheat–*Ae. comosa* genetic stocks

To produce wheat–alien cytogenetic stocks, the tetraploid durum wheat variety ’GK Novodur’ was pollinated with *Ae. comosa* accession MvGB1039 to produce F_1_ hybrids (Fig. 1). Pollination of 10 durum wheat spikes yielded a total of 101 durum wheat–*Ae. comosa* F_1_ seeds. Fourteen F_1_ plants were treated with colchicine, resulting in the production of 341 amphiploid (AABBMM) seeds. Five durum wheat–*Ae. comosa* amphiploid plants were subsequently top-crossed with the hexaploid wheat line Mv9kr1*ph1b*. The first top-cross produced 140 TC_1_ seeds. A second top-cross using 30 TC_1_ plants resulted in 482 TC_2_ seeds.

Finally, a total of 52 TC_2_F_1_ seeds representing different TC_1_-TC_2_ lineages were selected and analyzed by sequential GISH and FISH for the detection of M-genome and wheat chromatin.

### Frequency of M and D chromosomes

**Fig. 2:**
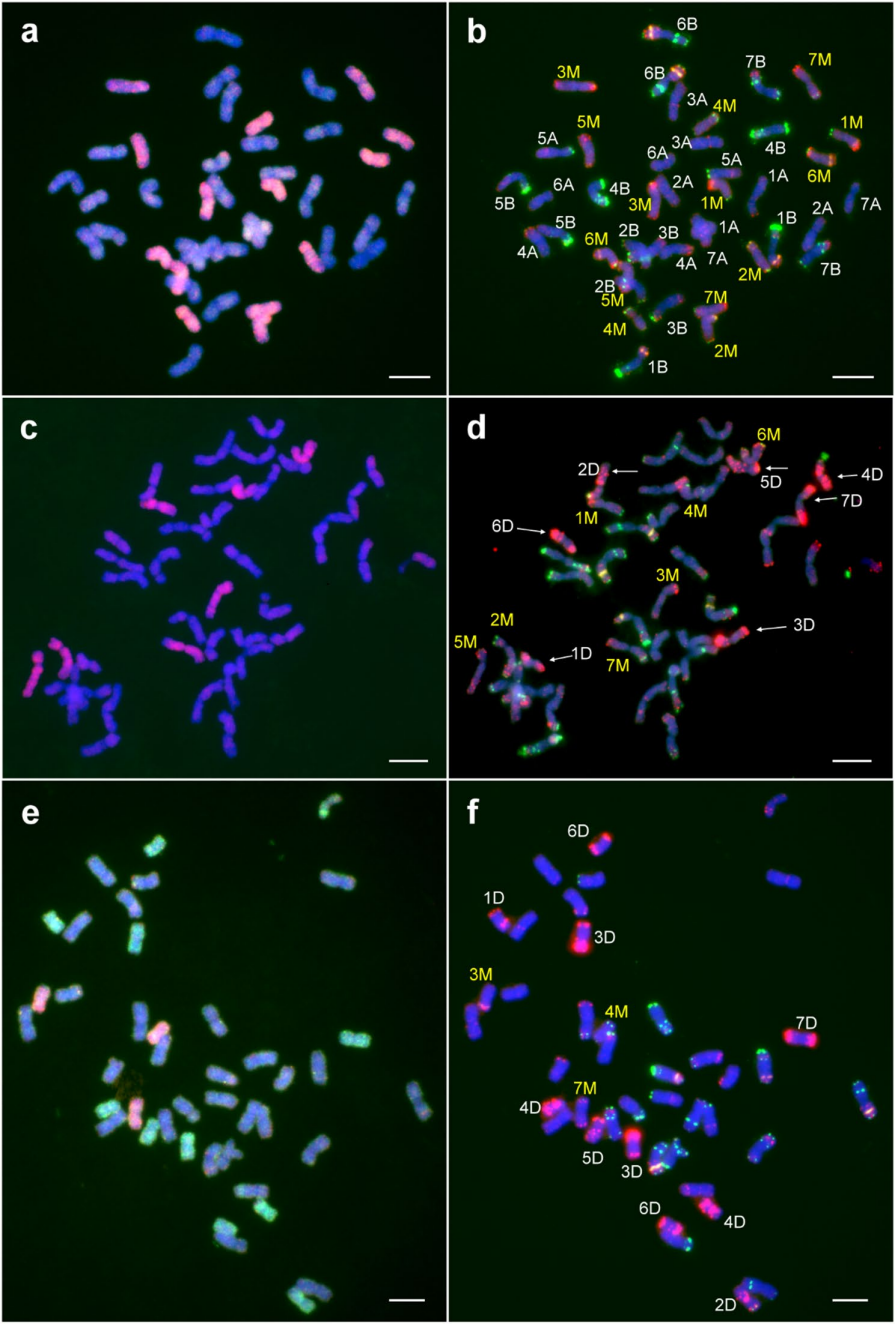
Representative mitotic metaphase cells of *T. turgidum* ssp. *durum* (‘GK Novodur’) – *Ae. comosa* (MvGB1039) amphiploid lines (**a**, **b**), their TC_1_ progenies developed by top-crossing with winter wheat line Mv9kr1*ph1b* (**c**, **d**), and TC_2_F_1_ lines (**e**, **f**). In the GISH images (**a**, **c**, **e**), the M chromosomes were visualized in red and the D chromosomes were visualized in green. The A and B chromosomes of Mv9kr1*ph1b* were counterstained by DAPI (blue). FISH (**b**, **d**, **f**) was carried out using the following DNA repeat probes: Afa-family (red), pSc119.2 (green), and pTa71 (yellow). Scale bar = 10 μm

The molecular cytogenetic analysis using GISH and FISH confirmed the expected genome composition of durum wheat–*Ae. comosa* synthetic hexaploid lines. All four analyzed amphiploid lines contained 14 A-genome and 14 B-genome chromosomes from wheat, along with 14 M-genome chromosomes from *Ae. comosa* (Fig. 2a, b). All seven M chromosomes (1M–7M) were successfully identified based on the hybridization patterns of the DNA repeat probes Afa-family, pSc119.2 and pTa71, as previously described for *Ae. comosa* accession MvGB1039 [50, 62]. The FISH patterns of the amphiploids also confirmed that the complete chromosome complements of the A-, B- and M-genomes were present in disomic form.

We also analyzed the genome composition of wheat–*Ae. comosa* TC_1_ lines (Fig. 2c, d). As expected, following the top-crossing with the hexaploid wheat line Mv9kr1*ph1b* (AABBDD), all seven M-genome chromosomes (1M–7M) were present in a single copy, as confirmed by the hybridization signals of the M-genome-specific probe. Similarly, D-genome chromosomes (1D-7D) were also detected in monosomic condition, as indicated by the specific hybridization pattern of the Afa-family probe. In contrast, the A- and B-genome chromosomes were in disomic form.

Following the second top-cross with hexaploid wheat Mv9kr1*ph1b*, we examined the transmission frequency of M-genome chromosomes in the selfed progeny (TC_2_F_1_) lines. In total, 156 M-genome chromosomes were detected by GISH across 52 lines, which corresponds to a theoretical expectation of 0.428 chromosomes per homoeologous group per line, assuming equal transmission (156/7/52 = 0.428). However, the FISH hybridization pattern of the 52 TC_2_F_1_ lines showed unequal transmission of M chromosomes (Fig. 2e, f; Table 1, Data S1). Chromosomes 1M and 6M were most frequently detected, present in 0.56 and 0.52 chromosomes per line, respectively. Moderate frequencies were observed for chromosome 4M (0.48), 2M (0.40), 3M (0.38), and 5M (0.35), while chromosome 7M exhibited the lowest transmission frequency (0.31). Chi-square (χ^2^) tests indicated that the observed deviations from equal transmission were statistically significant (*p* ≤ 0.05). In some TC_2_F_1_ lines, certain M chromosomes were observed in disomic form: 1M (7 lines), 2M (3 lines), 3M (2 lines), 4M (1 line), 6M (5 lines), and 7M (1 line) **(**Data S1**)**.

**Table 1:** Number and frequency (expressed as no. of chromosomes per line) of M-genome chromosomes in wheat–*Ae. comosa* TC_2_F_1_ population

M chromosome	1M	2M	3M	4M	5M	6M	7M	Total
No. of TC_2_F_1_ lines	52	52	52	52	52	52	52	
No. of M chrs.	29	21	20	25	18	27	16	156
Frequency	0.56*	0.40*	0.38*	0.48*	0.35*	0.52*	0.31*	0.43^#^
Chr. size^##^ (Mb)	557.6	644.8	693.8	574.0	619.0	574.8	698.5	

^#^Theoretical frequency for each M-genome chromosome in the case where the detected M chromosomes are evenly distributed among the seven homoeologous groups

^##^Data from Li et al. [64]

*Significantly different from theoretical frequency at *p* ≤ 0.05

Following the second top-cross with the hexaploid wheat line Mv9kr1*ph1b*, the complete D-genome chromosome complement was expected to be present in disomic form in the selfed progeny, such that two chromosomes per homoeologous group would occur in each line. However, GISH using a labeled *Ae. tauschii* genomic DNA probe, together with FISH employing Afa-family repeats, detected only 542 D-genome chromosomes across the 52 lines, instead of the expected 728 (52 × 14). Furthermore, the distribution of individual D-genome chromosomes was uneven. Chromosomes 1D, 2D, and 4D were underrepresented; although 1D and 4D are among the smallest D-genome chromosomes, 2D is the largest. In contrast, chromosomes 3D, 6D, and 7D were overrepresented relative to the theoretical frequency expected for an even distribution – 3D and 7D are large, whereas 6D is among the smallest (Table 2).

**Table 2:** Number and frequency (expressed as no. of chromosomes per line) of D-genome chromosomes in wheat–*Ae. comosa* TC_2_F_1_ population

D chromosome	1D	2D	3D	4D	5D	6D	7D	Total
No. of TC_2_F_1_ lines	52	52	52	52	52	52	52	
No. of D chrs.	72	69	79	75	77	88	82	542
Frequency	1.38^*^	1.33^*^	1.52^*^	1.44^*^	1.48	1.69^*^	1.58^*^	1.489^#^
Chr. size^##^ (Mb)	498.6	656.5	619.6	518.3	569.9	495.3	642.9	

^#^Theoretical frequency for each D-genome chromosome in the case where the detected D chromosomes are evenly distributed among the seven homoeologous groups

^##^Data from Zhu et al. [65]

^*^Significantly different from theoretical frequency at *p* ≤ 0.05

**Fig. 3:**
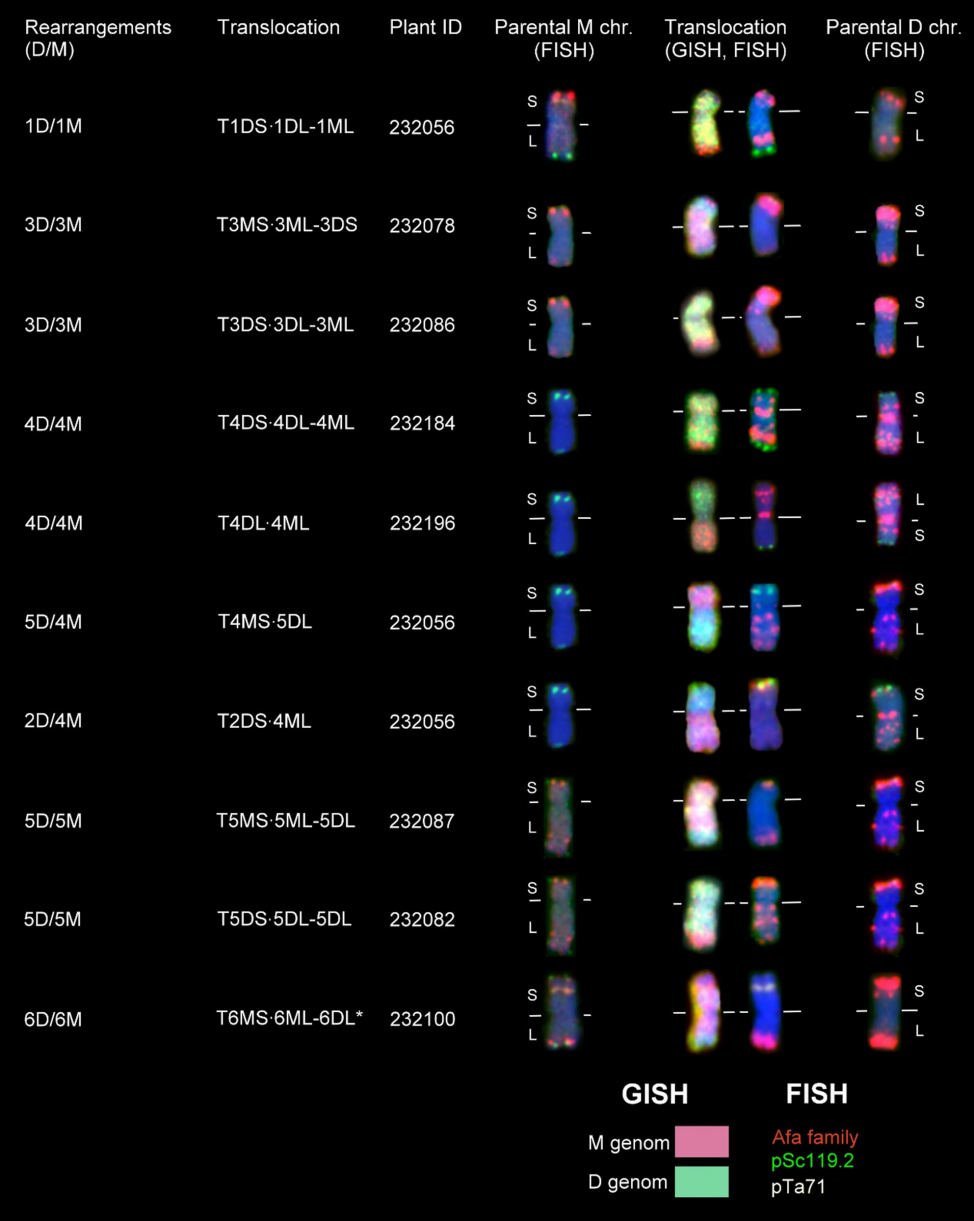
Molecular cytogenetic characterization of wheat–*Ae. comosa* (MvGB1039) D/M translocations detected in TC_2_F_1_ plants, and comparison of their FISH hybridization patterns with those of bread wheat (Mv9kr1*ph1b*) and *Ae. comosa* chromosomes. In the case of the translocated chromosomes, D-genome chromatin is visualized in green, while M-genome chromatin is visualized in red. FISH hybridization patterns were obtained using DNA repeat probes for the Afa-family (red), pSc119.2 (green), and pTa71 (yellow). The horizontal white line indicates the position of the centromere. S: short arm, L: long arm

Thirty-one wheat–*Ae. comosa* chromosomal rearrangements were also detected by GISH in the TC_2_F_1_ population, which were classified according to the wheat– and M chromosomes involved in the rearrangement as identified by the FISH hybridization pattern (Data S1; Table 3; Figs. 3 and 6). Twenty-eight intergenomic translocations out of the 31 rearrangements were homoeologous, as the wheat and *Aegilops* chromosomes involved in the translocations belonged to the same homoeologous group. In contrast, three translocations, two 5D/4M and one 2D/4M, were non-homoeologous. Theoretically, the 28 homoeologous wheat–*Aegilops* translocations should be distributed equally among the groups 1–7, with an expected mean of 4.0 translocations per homoeologous group. However, chi-square analysis showed significant deviation from this theoretical distribution. We found that the most frequent translocations occurred between chromosomes 5D and 5M (0.25). Other rearrangements, such as 1D/1M (0.077 translocations per line) and 6D/6M (0.096), as well as 3D/3M (0.077) and especially 4D/4M (0.038) were less common. Interestingly, intergenomic translocations between group 2 or group 7 wheat and *Ae. comosa* chromosomes were not detected (Table 3).

**Table 3:** Number and frequency (expressed as No. of rearranged chromosomes per line) of homoeologous wheat–*Aegilops* translocations in the TC_2_F_1_ population

	1D/1M	2D/2M	3D/3M	4D/4M	5D/5M	6D/6M	7D/7M	Total
Nr. of TC_2_F_1_ lines	52	52	52	52	52	52	52	
Number	4	0	4	2	13	5	0	28
Frequency	0.077	0	0.077	0.038^*^	0.250^*^	0.096^*^	0	0.076^#^

^#^Theoretical frequency for each D/M homoeologous translocation classes in the case where the detected D/M rearranged chromosomes are evenly distributed among the seven homoeologous groups

^*^Significantly different from theoretical frequency 7.69% at *p* ≤ 0.05

### Disomic wheat–*Ae. comosa* introgressions

**Fig. 4:**
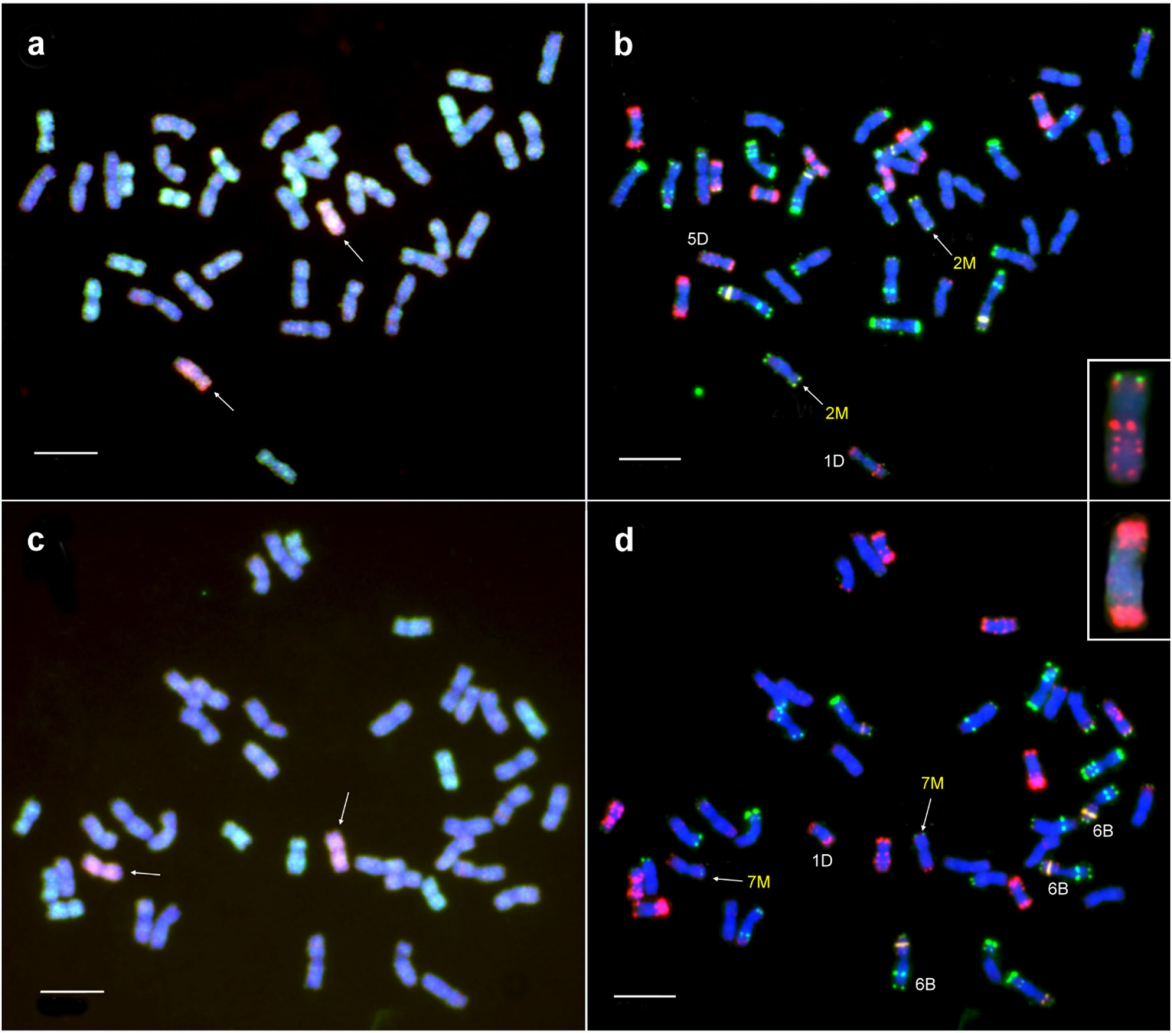
Partial mitotic metaphase cells of wheat–*Ae. comosa* disomic substitution lines. **a**-**b** Disomic 2M(2D) substitution line detected in the TC_2_F_1_ line 232,108 (Insert: the missing chromosome 2D). **c**-**d** Disomic 7M(7D) substitution line identified in the TC_2_F_1_ line 232,048 (Insert: the missing chromosome 7D). In the c and d images, the chromosomes in monosomic or trisomic forms are labeled by white letters. In the GISH images (**a**, **c**), the M chromosomes were visualized in red, the D chromosomes in green, while the unlabeled A and B chromosomes were counterstained by DAPI (blue). FISH was carried out using DNA repeat probes for Afa-family (red), pSc119.2 (green), and pTa71 (yellow). Scale bar = 10 μm

The main goal of the interspecific hybridization program is the production of disomic wheat–*Ae. comosa* germplasm lines with genetically stable inheritance. From this point of view, TC_2_F_1_ lines that carry only one homologous pair of M-genome chromosomes or a wheat–*Aegilops* translocation in the wheat genetic background are of particular importance. Using sequential GISH and FISH, we identified the submetacentric chromosome 2M characterized by telomeric pSc119.2 signals on both the short and long arms and weak subtelomeric Afa signals on both arms, and confirmed the absence of chromosome 2D in lines 232,108 and 232,111. We also found that wheat chromosomes 1D and 5D in line 232,108, and 1D, 3D, and 7D in line 232,111 were monosomic, consistent with their chromosome number of 40 and 39, respectively (Data S1). The chromosome composition indicates that these lines are promising sources for the production of a wheat–*Ae. comosa* 2M(2D) disomic substitution line (Fig. 4a, b).

We also identified the slightly submetacentric chromosome 7M characterized by telomeric pSc119.2 and Afa signals on the short and long arms, respectively, in disomic form, along with the absence of chromosome 7D in the 42 chromosome line 232,048. However, chromosomes 1D and 2D were monosomic, whereas chromosome 6B was trisomic in this 41- chromosome line (Fig. 4c, d).

**Fig. 5:**
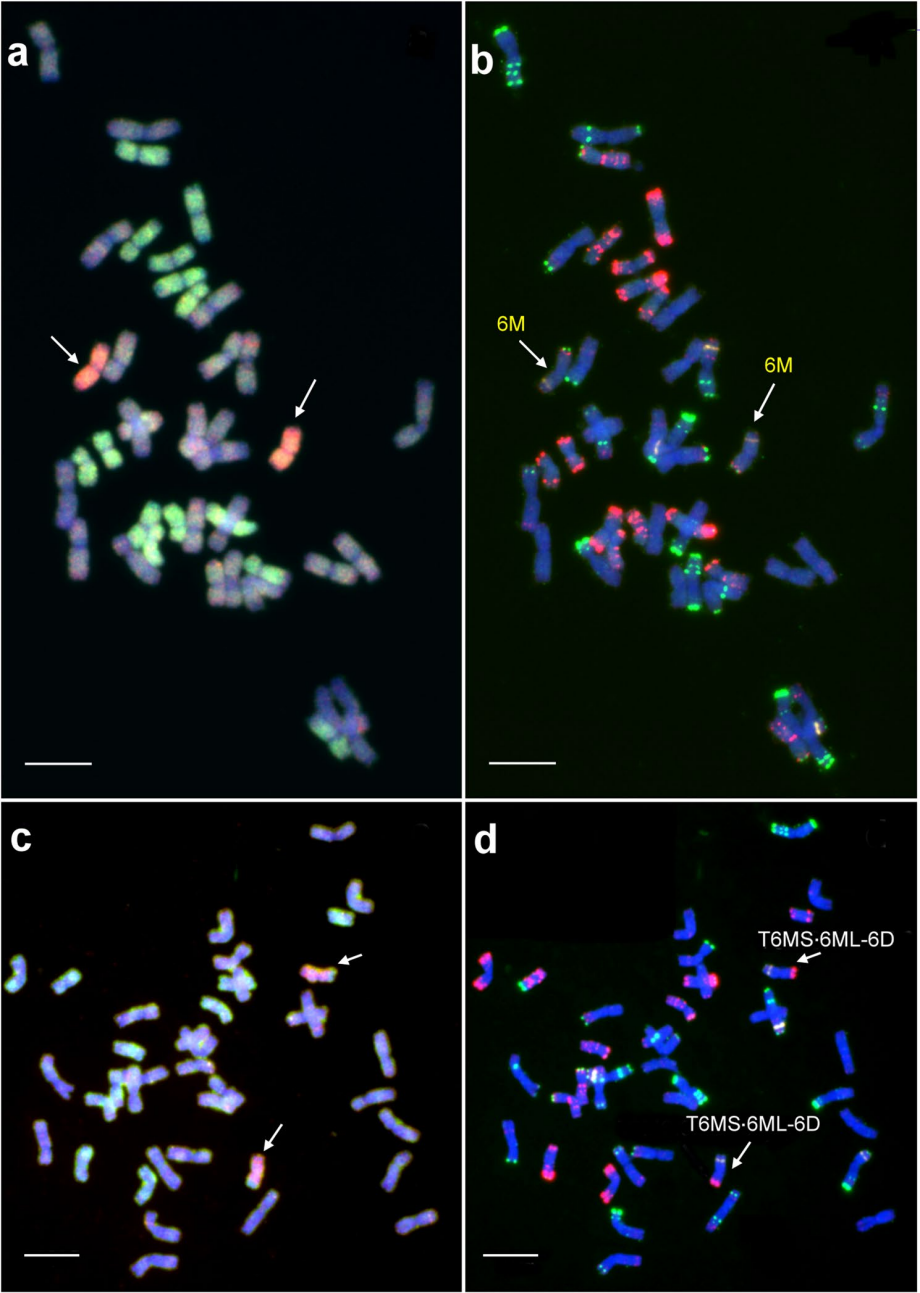
Detection of added chromosome 6M (**a**, **b**) and translocation T6MS·6ML-6D (**c**, **d**) in disomic form in Mv9kr1*ph1b–Ae. comosa* TC_2_F_1_ lines 232,042 and 232,100, respectively. In GISH images (**a**, **c**), the M chromosomes were visualized as red, while D chromosomes were visualized as green. The unlabeled A and B chromosomes were counterstained with DAPI (blue). FISH (**b**, **d**) was carried out using DNA repeat probes for Afa-family (red), pSc119.2 (green), and pTa71 (yellow). Scale bar = 10 μm

The line 232,042, with 44 chromosomes, contained a pair of satellited M-genome chromosomes, showing a weak telomeric pSc119.2 signal (not always visible) on the short arm, and definite pSc119.2 and Afa signals on the long arm at the telomeric and subtelomeric positions, respectively (Fig. 5a, b). This hybridization pattern corresponds to chromosome 6M, as the other satellited M-genome chromosome, 1M, has a strong telomeric Afa signal on the satellite and a strong pSc119.2 telomeric signal on the long arm. Finally, line 232,100, with 40 chromosomes, contained a disomic T6MS·6ML-6D translocation (Fig. 5c, d), identified by the strongly subtelomeric position of the secondary constriction and the absence of a visible pSc119.2 signal on the short arm. We also found that chromosomes 1D and 4D were monosomic in this line.

The chromosome composition indicates that these lines are promising sources for the development of wheat–*Ae. comosa* disomic substitution lines 2M(2D) and 7M(7D), a 6M disomic addition line, and a T6MS·6ML-6D disomic translocation line (Fig. 4a, b).

### Morphological traits and yield components of new wheat–*Aegilops* lines

**Fig. 6:**
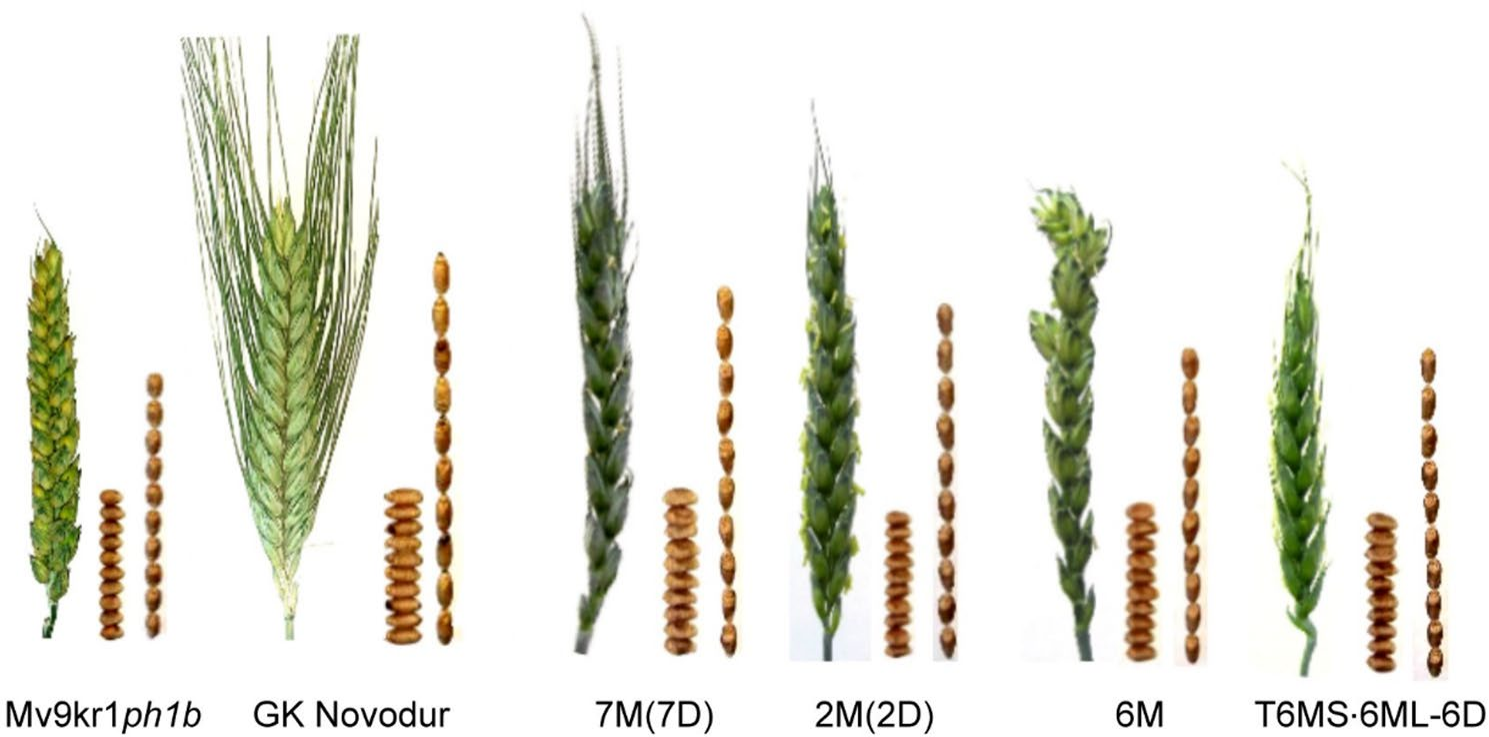
Spike and seed morphology of Mv9kr1*ph1b*, GK Novodur, Mv9kr1*ph1b–Ae. comosa* (MvGB1039) disomic 7M(7D) and 2M(2D) substitution lines, disomic 6M addition line, and disomic T6MS·6ML-6D translocation line

The morphological traits (plant and spike architecture) and yield components were examined under glasshouse conditions for the new wheat–*Ae. comosa* lines containing one homologous pair of M chromosomes (Fig. 6; Table 4). The 7M(7D) and 2M(2D) substitution lines, as well as the 6M addition line, exhibited greater height than the parental line Mv9kr1*ph1b*. Tillering (number of spikes per plant) and spike length of wheat–*Ae. comosa* lines did not differ significantly from the hexaploid wheat parent. It should be noted that most of the genotypes produced only one tiller. Beside the fact that the length of main spike was similar for the investigated genotypes with only the translocation line showing a shorter spike (7.5 cm), the number of spikelets per main spike was lower (~ 15–16) in the case of the substitution and addition lines, and especially the translocation line (13) in comparison with the parental tetraploid and hexaploid wheat genotypes (~ 20–21).

**Table 4:** Morphological traits of Mv9kr1*ph1b*, GK Novodur, and Mv9kr1*ph1b–Ae. comosa* (MvGB1039) disomic 7M(7D) and 2M(2D) substitution lines, disomic 6M addition line, and disomic T6MS·6ML-6D translocation lines grown in the glasshouse (2024, Martonvásár)

Genotype	Plant height (cm)	No. of spikes per plant	Length of main spike (cm)	No. of spikelets per main spike	No. of grains per main spike	Fertility (grains per spikelet)	TKW (g)
Mv9kr1 ph1b*	62 ± 7.9	1 ± 0.3	8 ± 0.9	20 ± 1.9	43 ± 5.4	2.15	36 ± 5.9
GK Novodur*	80 ± 6.8	1 ± 0.0	8.8 ± 0.5	21 ± 1.4	54 ± 1.8	2.57	45 ± 4.8
7M(7D)	70	2	9	15	17	1.13	40.52
2M(2D)	90	1	8.8	16	32	2.00	40.58
6M	80	1	9	15	46	3.06	39.74
T6MS·6ML-6D	60	1	7.5	13	23	1.76	37.21

*Data are means ± standard deviations of ten plants

In line with these differences, a lower number of grains per spike was observed in the substitution lines 2M(2D) and 7M(7D), as well as in the T6MS·6ML-6D translocation line. In contrast, the 6M addition line produced a comparable number of grains (46) relative to wheat parents (43 and 54 grains). However, fertility (expressed as the number of grains per spikelet) was similar in the 2M(2D) line and even higher in the 6M addition line compared to wheat lines (2.15 and 2.57, respectively). Finally, the thousand kernel weight of the 7M(7D) and 2M(2D) substitution lines, the disomic 6M addition, and the disomic T6MS·6ML-6D translocation line surpassed the average TKW of the hexaploid wheat parent Mv9kr1*ph1b.*

## Discussion

Double monosomic conditions for wheat and alien chromosomes in interspecific hybrid progenies facilitate the elimination of meiotic univalents, the formation of wheat–alien substitutions, the development of Robertsonian translocations through the centromeric breakage–fusion cycle, as well as the induction of homoeologous chromosome pairing and recombination during meiosis [66–68]. The gene transfer strategy based on top-crossing durum wheat–*Ae. comosa* amphiploids with hexaploid wheat exploited these effects, as the TC_1_ generation was monosomic for D- and M-genome chromosomes, thereby enabling the development of new wheat–*Ae. comosa* genetic resources within a short time. Our results showed that each homoeologous group M chromosome was transmitted to TC_2_F_1_ generation at a different rate, with chromosomes 1M, 6M and 4M being the most frequently retained, whereas the frequencies of 2M, 3M, 5M and 7M were below average. These results differ from the M chromosome transmission pattern of *Ae. biuncialis* — a natural allotetraploid of *Ae. comosa* and *Ae. umbellulata* — observed in hexaploid wheat–*Ae. biuncialis* backcrossed (BC_3_) populations by Gaál et al. [69]. In that study, the chromosomes 3M^b^, 4M^b^ and 5M^b^ were the most frequently retained, while the others were rare (1M^b^, 6M^b^), or absent (2M^b^, 7M^b^) in the populations. Depending on the degree of kinship, several mechanisms of chromosome elimination have been proposed in wheat interspecific hybrid progenies, including asynchronous cell cycles between the wheat and alien genomes [70], altered spindle attachment to different chromosome sets, as observed in wheat × maize zygotes [71], and more recently, the non-functionalization of centromeres caused by improper incorporation of CENH3 due to the malfunction of proteins involved in its loading and assembly [72, 73].

However, these mechanisms usually operate at the early steps of alien gene transfer, resulting in the complete elimination of the uniparental chromosome complement and haploidization already in the F_1_ hybrid plants [73]. The fact that the full set of M-genome chromosomes was present in both the durum wheat–*Ae. comosa* synthetic hexaploids and TC_1_ generation suggest that the differences in M chromosome frequencies observed in the TC_2_F_1_ populations cannot be attributed to centromere malfunction. The proper assembly of a functioning *Ae. comosa* centromeres in the wheat genetic background is further supported by the close phylogenetic relationship between the genomes of *Ae. comosa*, durum and bread wheat [74].

This close phylogenetic relationship has also been manifested in the similar genome structure of the M- and D-genomes. Comparative genome analysis based on the mapping of conserved orthologous genes using PCR markers [51, 62] and single-gene FISH [50], as well as the high-resolution segregating genetic map of *Ae. biuncialis* M^b^-genome chromosomes [75] and the chromosome-scale reference genome of *Ae. comosa* [64], confirmed that the M-genome preserves significant macro-collinearity with the corresponding D-genome chromosomes of hexaploid wheat. Consistent with the high level of D–M genome synteny, frequent M^b^–wheat chromosome associations have been reported during meiotic metaphase I of wheat x *Ae. biuncialis* F_1_ hybrids [54]. Besides the elimination of M-genome chromosomes, we also detected the unequal elimination pattern of D chromosomes in TC_2_F_1_, as 1D, 2D and 4D were eliminated with higher frequency, while chromosomes 5D and 3D, 6D and 7D were eliminated with equal and lower frequency than the mean, respectively. The elimination patterns of M and D-genome chromosomes seem to be related, to some extent, to chromosome size. Flow cytometric analysis, microscopy measurements of mitotic chromosomes, and whole F sequencing studies showed that the frequently retained chromosomes 1M, 4M, and 6M are smaller (6.53–7.04 μm, 557.6–574.8.6.8 Mb) than the frequently eliminated 2M, 3M, 5M, and 7M chromosomes (7.58–7.78 μm, 619.0–698.5.0.5 Mb) [50, 64]. Interestingly, an opposite trend emerged for D chromosomes, as the chromosomes 1D and 4D, frequently eliminated in our study, are smaller (8.4 μm and 9.0 μm, respectively) than the retained chromosomes 3D, 7D, and 5D (10.7 μm, 10.1 μm and 10.4 μm) [76]. However, we note that the largest D-genome chromosome, 2D, was also eliminated at a high frequency.

According to a recent hypothesis regarding genome stability in wheat × alien hybrid derivatives [77], the size of the introgressed chromosomes may influence their elimination frequency through their positioning in the 3D-space of interphase nuclei, where individual chromosomes occupy specific territories [78]. In Rabl’s configuration of chromosome territories, characteristic of wheat and other *Triticeae* species [79, 80], centromeres and telomeres form two distinct clusters at opposite poles of the interphase nucleus [81]. Using hexaploid wheat lines carrying chromosomes and chromosome arms introgressed from rye or barley to investigate the 3D structure of somatic interphase nuclei, Koláčková et al. [77] observed a tendency for shorter chromosomes and arms to be located in the nuclear interior, whereas longer ones were often found near the nuclear periphery. In a subsequent study, the authors suggested a direct connection between the nuclear positioning of introgressed chromosomes and their reduced ability to migrate into the telomere clusters at the onset of meiosis [82], which reduces the likelihood of probability at metaphase I [83, 84], ultimately resulting in the gradual loss of the introgressed chromosomes. In the present study, the TC_1_ generation contained the complete set of D-genome chromosomes and structurally similar M-genome chromosomes in monosomic form. It is conceivable that the smaller M chromosomes (1M, 4M, and 6M) had a greater chance of occupying appropriate positions within the interphase nucleus and migrating into the telomere bouquet by displacing D chromosomes. This could facilitate the formation of homoeologous M(D) substitutions and recombinations in TC_2_F_1_ generation. Due to the high level of M-D chromosome synteny and shared gene content, the presence of M chromosomes compensates well for the loss of D chromosomes, as suggested by the maintained fertility. However, further studies using 3D-FISH are needed to compare the spatial positioning of M- and D-genome chromosomes in nuclei during different stages of the vegetative and generative cell cycles. The frequent elimination of the large chromosome 2D indicates that chromosome size alone does not account for chromosome elimination.

Chromosome elimination can also result from chromosomal breaks induced by gametocidal genes, which are frequently observed in *Aegilops* species. Gametocidal genes have been reported on chromosomes 4M^g^ and 4M^b^ of the allotetraploid *Ae. geniculata* and *Ae. biuncialis* [85–87] and it cannot be excluded that chromosome 4M of their diploid progenitor, *Ae. comosa*, also has a gametocidal effect. This hypothesis appears to be supported by the non-homoeologous 5D/4M and 2D/4M wheat–*Ae. comosa* translocations were detected in the present study. On the other hand, the higher frequency of homoeologous chromosome rearrangements between group 1, 3, 4, 5, and 6 chromosomes suggests that chromosomal synteny between wheat and *Ae. comosa* may have a more dominant role in promoting meiotic chromosome pairing and recombinations. In this context, chromosome 5M may harbour a *Ph1*-suppressor locus facilitating homoeologous recombination, as reported for chromosome 5M^g^ of *Ae. geniculata* [88].

Another objective of our research was to develop new wheat–*Ae. comosa* genetic stocks, including additions, substitutions, and translocation lines. The TC_2_F_1_ generation contained all *Ae. comosa* chromosomes (1M–7M), and the resulting introgression lines may carry novel gene variants with potential agronomical benefits. Chromosome 1M of *Ae. comosa* has been reported to have a positive effect on seed morphology and bread-making quality traits [44, 45]. Similarly, Garg et al. [89] demonstrated that chromosome 1M^g^ of *Ae. geniculata* positively affects seed volume–weight, protein content, grain hardness, sedimentation value, and gluten content. Chromosome 2M of *Ae. comosa* carries several resistance genes against fungal diseases (*Lr57*, *Sr34*, *Sr53*, *Yr8*, *Yr40*) [90], but it has also been linked to reduced grain width, plant height, spikelet number, grain number, and thousand kernel weight. Chromosome 3M increases internode elongation between spikelets, yet it likewise reduces flag leaf width, spikelet number, and grain number per spike [32]. Rakszegi et al. [27] showed that group 2 chromosomes from *Ae. geniculata* and *Ae. biuncialis*, as well as chromosome 3M^b^, enhance grain protein content in wheat–*Aegilops* addition lines. Additionally, several QTLs conferring resistance to powdery mildew (race E09) and stripe rust (races CYR31–CYR34) have been identified on chromosome 3M^g^ [91]. Liu et al. [32] reported that chromosome 4M reduces plant height, flag leaf width, the number of spikelets per spike, and the number of grains per spike. The 4M^b^·4BS centric fusion line exhibited significantly higher zinc and manganese content compared to bread wheat [29, 69]. As mentioned previously, chromosomes 4M^g^ and 4M^b^ carry gametocidal genes [85–87]. Chromosome 5M has also been shown to reduce plant height, flag leaf width, spikelet number, and grain number per spike, while its homoeologue, 5M^g^, contributes to increased grain protein content. Similarly, chromosome 6M decreases flag leaf width, number of spikelets per spike and number of grains per spike [32]. *Ae. comosa* carries a QTL responsible for powdery mildew resistance located on chromosome 7M [32], and both chromosomes 7M^b^ and 7M^g^ have been associated with higher grain protein content [27]. The chromosome segments introgressed from *Ae. comosa* accession MvGB1039 into hexaploid winter wheat Mv9kr1*ph1b* represents potentially important gene complexes for the wheat breeding programs. However, it should be noted that before using these genetic stocks in elite breeding programs, the *ph1b* mutant chromosome 5B must be eliminated by backcrossing these lines with the wild-type Mv9kr1*ph1b* to stabilize the genome.

It is also important to emphasize that most of the wheat–*Aegilops* intergenomic translocations are in monosomic form, therefore, the selection of disomic translocation lines is required to ensure stable inheritance and enable reliable assessment of the effects of alien chromatin on wheat agronomic traits.

## Conclusion

Analysis of the transmission frequencies of individual M- and D-genome chromosomes demonstrated that top-crossing durum wheat × *Ae. comosa* amphiploids with hexaploid wheat is an effective strategy for developing wheat–*Ae. comosa* introgression lines. Monosomic conditions in the two genomes promoted the elimination of both M- and D-genome chromosomes, although chromosome size may have influenced their retention. In general, shorter M chromosomes (1M, 4M, 6M) were preferentially maintained, whereas shorter D chromosomes (1D, 4D) were predominantly eliminated, leading to M(D) substitutions and intergenomic translocations. Beyond chromosome size, additional factors—such as the presence of gametocidal loci or the wheat–*Aegilops* homoeologous relationships—may also contribute to differential chromosome elimination. Monitoring chromosome transmission and rearrangement across generations will support the development of novel and agronomically valuable wheat–*Ae. comosa*introgressions.

## Supplementary Information

Supplementary Material 1. Data S1. Number of M- and D-genome chromosomes observed in wheat–*Ae. comosa* TC_2_F_1_ generation, and the number of wheat–*Aegilops* homoeologous recombinations.

## Data Availability

The datasets supporting the conclusions of this article are included within the article and its additional files.

## Abbreviations

FISH: Fluorescence in situ hybridization
GISH: Genomic in situ hybridization
TC: Top-crossed
MvGB: Martonvásár Gene-Bank
DAPI: 4’-6’-diamidino-2-phenylindole
*Ae. comosa*: *Aegilops comosa*
